# Need for Space: The Key Distance Effect Depends on Spatial Stimulus Configurations

**DOI:** 10.1371/journal.pone.0091432

**Published:** 2014-03-18

**Authors:** Melanie Jonas, Owino Eloka, Julia Stephan, Volker H. Franz

**Affiliations:** Department of Psychology, University of Hamburg, Hamburg, Germany; McGill University, Canada

## Abstract

In numerous psychological experiments, participants classify stimuli by pressing response keys. According to Lakens, Schneider, Jostmann, and Schubert (2011), classification performance is affected by physical distance between response keys – indicating a cognitive tendency to represent categories in spatial code. However, previous evidence for a key distance effect (KDE) from a color-naming Stroop task is inconclusive as to whether: (a) key separation automatically leads to an internal spatial representation of non-spatial stimulus characteristics in participants, or if the KDE rather depends on physical spatial characteristics of the stimulus configuration; (b) the KDE attenuates the Stroop interference effect. We therefore first adopted the original Stroop task in Experiment 1, confirming that wider key distance facilitated responses, but did not modulate the Stroop effect as was previously found. In Experiments 2 and 3 we controlled potential mediator variables in the original design. When we did not display instructions about stimulus-response mappings, thereby removing the unintended spatial context from the Stroop stimuli, no KDE emerged. Presenting the instructions at a central position in Experiment 4 confirmed that key separation alone is not sufficient for a KDE, but correspondence between spatial configurations of stimuli and responses is also necessary. Evidence indicates that the KDE on Stroop performance is due to known mechanisms of stimulus-response compatibility and response discriminability. The KDE does, however, not demonstrate a general disposition to represent any stimulus in spatial code.

## Introduction

Almost any undergraduate student of Psychology has earned course credits by completing one variation or another of the following task: identifying the color of a visual stimulus, while ignoring the meaning of a color word that itself carries the color or that is presented at another time or position in the stimulus array. The observation that identification takes longer when the physical color and the meaning of the color word are incongruent than when both match has been named ‘Stroop effect’ after its discoverer John Riley Stroop [1]. Based on a vast number of replications hardly any other finding in psychology is as well-corroborated as the Stroop effect; for a review see [2].

### 1. An interesting effect of key distance on Stroop performance

Recently, Lakens, Schneider, Jostmann, and Schubert [3] reported an interesting finding from a two-choice key-press version of the Stroop task. They tested whether the spatial distance between response keys on a computer keyboard influenced the performance in this task. Participants were asked to indicate the color (blue or red) of a letter string (the Dutch words ‘blauw’ and ‘rood’ for ‘blue’ and ‘red’, or ‘XXXX’ as neutral string) by pressing either a left or a right key, while ignoring the meaning of the word. Color and meaning of the word could be congruent or incongruent. Surprisingly, they did not only find the well-known Stroop interference effect. Also, average reaction time (RT) was shorter when participants used the response keys that were located far apart on the keyboard, compared to when keys were close together (approximately 33 ms). This key distance effect (KDE) was more pronounced in incongruent trials (close-far  =  61 ms) than in congruent (15 ms) or in neutral trials (22 ms). In their replication (Experiment 1), Proctor and Chen [4] obtained a similar RT pattern.

The KDE on Stroop performance is of general interest for two reasons: (a) the effect contradicts expectations based on earlier findings on the influence of key separation in experiments where both stimuli and responses have a spatial dimension, given that in the Stroop task there is no obvious correspondence between spatial characteristics of responses and stimuli. (b) The KDE might have far-reaching implications for cognitive psychology – because it might indicate a general cognitive tendency to use spatial code for mental categories.

### 2. Key distance and spatial stimulus-response compatibility

What makes the demonstration of the KDE so surprising in the first place is that in the Stroop task there is no correspondence between the spatial characteristics of the response configuration (left-right) and any feature of the stimuli. The task-relevant stimulus dimension of font color neither has imminent spatial features, nor are there obvious conceptual metaphors which might map the colors red and blue onto any spatial dimension shared with the response configuration, unlike, for example, numbers that are believed to be represented on a horizontal number line [5]. The letter strings also bear no spatial features, neither with regard to their meaning, nor their position on the screen, as stimuli were presented centrally at fixation.

In other paradigms, where stimuli and responses share spatial characteristics, effects of spatial stimulus-response (SR) compatibility have been repeatedly demonstrated: responses are usually faster and more accurate when the positions of stimulus and response coincide than when they do not. Even when the spatial position of a stimulus is not the task-relevant dimension, but participants have to respond to a non-spatial stimulus attribute, their spatial responses are nonetheless affected by the location of the stimulus, a phenomenon widely known as the ‘Simon’ effect [6]. According to Wallace [7], spatial SR compatibility is due to a comparison of spatial codes for stimulus and response positions, leading to a longer RT when representations of stimulus and response positions do not coincide.

To our knowledge, effects of response key separation in a Stroop paradigm have only been reported so far related to the basic design by Lakens et al. [3], which was adopted by Proctor and Chen [4], and recently by Nett and Frings [8]. However, an influence of key distance was found in earlier experiments with both spatial responses and visuo-spatial stimuli [9]–[11]. In fact, effects of key separation might depend on shared spatial characteristics of stimuli and responses.

In a response-cuing task by Miller [9] participants responded to the position of an asterisk on the screen by pressing one of four keys on a computer keyboard. Stimulus and response configuration corresponded in terms of their diamond-like spatial arrangement. It turned out that visual pre-cuing of responses was more effective in terms of accelerating RTs when response keys were located far apart at the left and right edges of the keyboard compared with keys close together at the center of the keyboard. Miller [9] suggested that response preparation primarily depends on a match between spatial cuing information and a spatial response code.

Further findings on the influence of key distance might also be due to spatial SR compatibility: Heister, Schroeder-Heister, and Ehrenstein [10] had participants make either spatially compatible or incompatible responses to right and left-lateralized visual stimuli. Participants responded with two fingers of either their left or right hand (different sessions) placed on different sets of two buttons each, with buttons separated by different horizontal distances (45 vs. 110 mm). The compatibility effect was significantly smaller when the buttons were farther apart (incompatible – compatible  =  26 ms) than when they were closer together (51 and 46 ms, depending on which combination of fingers was used).

Stins and Michaels [11] studied participants pressing mouse keys (Experiment 3), responding either spatially compatible or incompatible to the right or left half of six horizontally arranged visual stimuli. Mouse keys were actuated by the left or right index finger and separated horizontally by approximately 15 or 65 cm. While key separation did not modulate the effect of compatibility, it interacted with stimulus eccentricity. With wider separated keys, responses to more eccentric stimuli were faster than to more central stimuli. When participants carried out reaching movements to targets indicated by the horizontal stimuli instead of key-presses (Experiments 1 & 2), movement onset was always faster when the stimuli and the targets were both at central locations or both at eccentric locations (i.e., both close together or farer apart).

Adam, Hommel, and Umiltà [12] found that spatial compatibility of stimuli and responses affected key-presses in a response-cuing task (Experiments 2 & 3) modified after Miller [9]. Responses with fingers from both hands were faster and more accurate when both the horizontal configuration of four possible stimulus positions and four response positions was either a left-right pattern (with the two left and right positions close together) or an inner-outer pattern (with the two inner positions close together). This compatibility effect was found also when participants used fingers of one hand, however, only when response preparation was short.

Unlike in the above studies, in the Stroop task employed by Lakens et al. [3] and Proctor and Chen [4], spatial compatibility between Stroop stimuli and responses is not explicitly manipulated, rendering a KDE unexpected.

### 3. Potentially strong implications of the KDE on Stroop performance

Lakens et al. [3] proposed that the perceived distance between response keys automatically prompted participants to represent the colors of the Stroop stimuli in spatial codes, as an instance of a general tendency to organize mental categories in space. The authors' interpretation dates from the theoretical framework of extended cognition, the basic idea of which is the incorporation of the outer world into internal representations [13]. With respect to space in particular, Clark [13] primarily stated that it can be utilized to aid mental processing. Lakens et al. [3], moreover, argued that perceiving the response keys in the Stroop task as located far apart in extrapersonal space also spontaneously increases the mental space between representations of the stimulus categories (e.g., the colors). Mental separation of stimulus categories, in turn, facilitated selection of the correct response – all the more in the demanding incongruent trials where response selection was more difficult through interference from the word meaning. If however, as concluded by Lakens et al. [3], due to a general principle of human cognition key separation automatically affects mental representations, this would have a large impact on cognitive psychology. One would expect potentially any spatial arrangement of response keys to affect categorization of virtually any kind of stimulus.

Proctor and Chen [4] proposed a less general account: they assumed that the physical separation of response keys, or probably also other factors that distinguish the keys on the standard keyboard, enhances spatial discriminability of responses in the far key condition relative to the close key condition, thereby facilitating response selection with far keys. They furthermore consider the influence of key separation to be restricted to only the initial trials of an experiment (i.e., about 30 per condition). Their account of the KDE has therefore much less far-reaching implications than Lakens et al. 's [3].

The evidence we present here argues against Lakens et al. 's [3] strong interpretation of the KDE. Our findings furthermore imply that the KDE is not due to discriminability of responses alone, but rather to characteristics of both responses and stimuli.

### 4. Open questions

**Figure 1:**
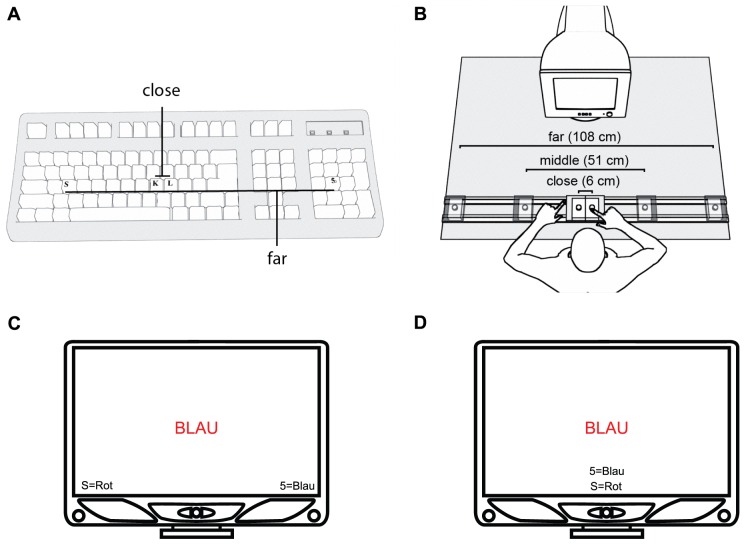
Response and stimulus set-ups in the different experiments. A. Experiments 1 and 3–4, after [3]: keyboard keys assigned to responses in the close (‘K’, ‘L’) and far (‘S’, ‘5’) distance conditions, respectively. B. Experiment 2: custom-made response keys were not labeled and could be adjusted in distance. C. Experiment 1: instructions about mappings of font colors to response keys were presented in the lower left and right corner of the screen. D. Experiment 4: instructions were presented centrally at the bottom of the screen.

We identify two relevant issues in the evidence on the KDE on Stroop performance that are worth a thorough reinvestigation: the first one regards a distinctive feature of the Stroop paradigm employed by Lakens et al. [3], Proctor and Chen [4] (Experiment 1), and Nett & Frings [8]. While the stimuli themselves did not convey any spatial information, instructions about SR mappings were presented as text at the bottom of the screen throughout all experiments, constantly reminding participants of which key they had to press in response to either color. Lakens et al. [3] positioned the SR mapping instructions in the lower left and right corner of the screen, spatially compatible with the positions of the respective keys. In the far condition, for example, ‘S  =  red’ was displayed in the lower left part of the screen, and ‘5  =  blue’ in the lower right part (see set-up of our Experiment 1, Fig. 1C). Presenting the SR mapping instructions in this way is rather unusual in RT experiments. Lakens et al. [3] largely adapted the study design of a Stroop experiment conducted by Jostmann and Koole [14] (who, however, did not manipulate key distance in their study). Displaying SR mapping instructions in a spatial configuration on the screen might bias participants towards encoding the two stimulus colors on the spatial dimension. Therefore, a KDE might not just arise because physical key separation leads to differences in discriminability of responses, but ultimately because the configuration of the SR mapping instructions on the screen shares the spatial dimension with manual responses. If this is the case, the previous Stroop studies would not provide conclusive information whether the KDE arises automatically from a general tendency to represent mental categories in space.

Secondly, previous evidence is still inconclusive as to whether perceived distance between response keys affects Stroop performance in general, or specifically facilitates responses in the incongruent condition, thereby attenuating Stroop interference: in accord with Lakens et al. [3], Proctor & Chen's [4] replication (Experiment 1) showed a significant interaction between key distance and Stroop congruency which was due to less Stroop interference with far (5 ms) than with close keys (51 ms). In their two further Experiments 2 & 3 [4], there was no significant interaction. In one of these, that is, Experiment 2 [4], a main effect of key distance was found. The interpretation of this effect remains unclear, because key distance was introduced as a between-subjects factor, including a third condition where subjects responded on close keys with fingers from a single hand, and no paired comparison between the two conditions with both hands was provided. In Proctor & Chen's [4] Experiment 3 key distance had no significant effect on RT at all. Nett and Frings [8] obtained both an interaction and a main effect of key distance in their conceptual replication (Experiment 1a). Despite analyses of variance (ANOVAs) showed different results, the numerical RT pattern was similar in all experiments, exhibiting faster responses with far than with close keys and a smaller Stroop effect with far keys.

The overall picture is, however, even more complicated. The above pattern was obtained only when at most 30 trials per key distance were analyzed in an experiment [3], [8], [4] (Experiment 1). When Proctor and Chen [4] analyzed all 720 trials from their longer experiments, the interaction effect vanished. Furthermore, mean RT was the same with close and far keys in Experiment 2, but tended to be slower with close keys in Experiment 3. Therefore, Proctor and Chen [4] assumed an influence of time-course or practice. This, however, would seem inconsistent with Lakens et al. 's [3] idea of an automatic effect on internal representation.

Further evidence is needed to resolve these inconsistencies. This is all the more relevant, as a persistent modulation of Stroop interference through key separation might provide decisive new insight in the origin of the Stroop effect itself. Following Proctor & Chen's [4] reasoning, if key distance specifically facilitates response selection in incongruent Stroop trials by reducing interference from semantic information on the color categorization task, this would indicate that Stroop interference cannot only result from stimulus-stimulus compatibility, but also from SR compatibility [15].

However, our present results suggest a key role of the displayed SR mapping instructions in the mediation of the KDE on Stroop performance. Furthermore, current evidence altogether does not corroborate a consistent attenuation of Stroop interference by key separation.

### 5. Our experiments

We addressed the above issues by systematically modifying Lakens et al. 's [3] original Stroop task in four experiments. In Experiment 1, using a design similar to Lakens et al. [3] (Fig. 1A, C), we probed the replicability of the KDE. Our results confirmed an influence of horizontal separation of response keys. RTs were significantly faster in total with far than with close keys. The Stroop effect was smaller when keys were close; however, the interaction was not significant. In Experiment 2 we removed potentially mediating factors for KDEs, other than physical key separation alone, from the configuration of stimuli and responses, increased the accuracy of the measurement, and tested a wider range of key distances (Fig. 1B). As we did not find a KDE, we returned to the original design by Lakens et al. [3] to identify the source of KDE by successively manipulating putative mediators. In Experiment 3 we removed the SR mapping instructions that had been displayed in a spatial configuration on the screen along with the stimuli in the original study (Fig. 1C). Because omitting the instructions also cancelled the KDE, we hypothesized that it was essentially driven by the spatial correspondence between the displayed SR mapping instruction and the actual responses. We tested this idea by reducing the correspondence between SR mapping instructions and responses with far keys. When we moved the instructions to the center of the screen in Experiment 4 (Fig. 1D), the KDE disappeared. Our findings show that the KDE reported by Lakens et al. [3] depends on spatial stimulus-response compatibility and response discriminability, but does not indicate a cognitive principle of spatial representation.

## Experiment 1

The goal of the first experiment was to replicate the KDE using methods analogous to Lakens et al. [3] and Proctor and Chen [4] in our laboratory. We used the same experimental task and set-up, especially an ordinary keyboard, and displayed SR mapping instructions on the screen throughout the task. Because of our German-speaking participants, we had to present the color words in German instead of Dutch. In place of a QWERTY keyboard we employed a QWERTZ keyboard with which our participants were more familiar. Moreover, we chose to present a somewhat more reliable number of 20 repetitions per Stroop condition (congruent, incongruent, neutral) instead of the very small number of 10 repetitions used in the earlier studies. Under conditions as in previous studies, we expected key distance to affect performance, either by way of general response facilitation with wider key separation, or by a specific reduction of RTs in incongruent trials in the far key condition.

### 1. Methods

#### 1.1 Participants

Twenty participants took part in Experiment 1 (18 females; age 18–37 years, *M*  = 22.6). In all experiments, participants were either undergraduate students who received course credits or paid volunteers. All were native German speakers, right-handed and had normal or corrected-to-normal vision by self-report.

#### 1.2 Ethics statement

Written informed consent was obtained from all participants. All experiments were conducted in accordance with the 1964 Declaration of Helsinki and with the ethical guidelines of the German Psychological Society (DGPs) and the Professional Association of German Psychologists (BDP) (2005, C.III). The study was conducted within the International Graduate Research Group “Cross-modal Interaction in Natural and Artificial Cognitive Systems” (CINACS) that was reviewed and approved by the German Research Foundation (DFG, project number IGK-1247) which did not require further Institutional Review Board approval.

#### 1.3 Apparatus, stimuli and procedure

Participants sat in a quiet room at a distance of approximately 60 cm from the screen. The Psychophysics Toolbox [16], [17] in Matlab (R2010b; The Mathworks, Inc., MA, USA) was used to control stimulus presentation on a Fujitsu Siemens CRT color monitor (screen diagonal: 43.2 cm, resolution: 1024×768 pixels, refresh rate: 100 Hz). Experimental stimuli were the German words ‘BLAU’ and ‘ROT’ for ‘blue’ and ‘red’, or the letter string ‘XXXX’ in uppercase Arial font. The strings were presented centrally on the screen, in red or blue on a white background (*x, y, Y* coordinates, with *x, y* being chromaticity coordinates within the CIE 1931 color space, and *Y* denoting luminance in cd/m^2^: red  = 7.581, 0.346, 81.7; blue  = 0.158, 0.93, 13.2, white  = 0.290, 0.316, 80.8). Keys on a standard QWERTZ keyboard were assigned to responses in the close (‘K’, ‘L’) and the far (‘S’, ‘5’) distance condition, respectively (Fig. 1A). Responses were made with the left or right index finger. SR mapping instructions were presented as text in the left and right lower corners of the screen throughout the task (e.g., ‘S  =  red’ to the left and ‘5  =  blue’ to the right; Fig.1c). Participants were instructed to identify as fast as possible the color of the centrally displayed stimulus by pressing the assigned response key, while ignoring the word meaning. Participants underwent 120 trials in computer-generated pseudo-random order in 2 blocks (close, far) of 60 trials each (20 per Stroop congruency: congruent, incongruent, neutral). Within participants, one of two SR mapping instructions (blue  =  right key or blue  =  left key) was used in both blocks, and instructions were counterbalanced across participants. Each block was preceded by 10 practice trials. A single trial started with a white screen presented for 200 ms, after which a black fixation cross was displayed in the center of the screen for 500 ms (*x, y, Y* CIE coordinates for black  = 0.366, 0.392, 3.25). Thereafter the target word appeared for 2000 ms, during which the response was made. In the practice trials, visual feedback (‘Falsch’, German word for ‘wrong’) was displayed for 500 ms in the center of the screen if an incorrect response was made. If participants failed to respond within 2000 ms in any trial, it was repeated at a random later time.

#### 1.4 Data analysis

Error trials were excluded from subsequent analysis on RT. We performed a 3 (Stroop congruency: congruent, incongruent, neutral) ×2 (key distance: close, far) repeated-measures ANOVA on mean RT and error rate. To correct for possible non-sphericity of data, Greenhouse-Geisser-adjusted *p*-values are given for all effects. Departure from sphericity is denoted as Greenhouse-Geisser-ε. Effect size is reported as η_p_^2^ [18] for F-tests and as Cohen's *d* [19] for t-tests. Considering repeated measures, *d* was estimated as 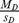. 95% confidence intervals are given for the mean difference between two conditions (i.e., Stroop effect  =  incongruent-congruent, main effect of key distance  =  close-far, interaction effect between Stroop congruency and key distance  =  Stroop effect for close keys-Stroop effect for far keys). Significance level was set to *α* = .05.

To assure that our main analysis would not miss any transient effect of key distance that is evident only in the initial trials, we followed the procedure of Proctor and Chen [4] by additionally analyzing the first 30 trials per key distance (i.e., 10 trials per Stroop congruency). If not stated otherwise, the same analyses are reported for all experiments. The raw data of our experiments can be openly accessed via the Open Science Framework (https://osf.io/7gj3u/).

### 2. Results

#### 2.1 Analysis of all 120 trials

**Figure 2:**
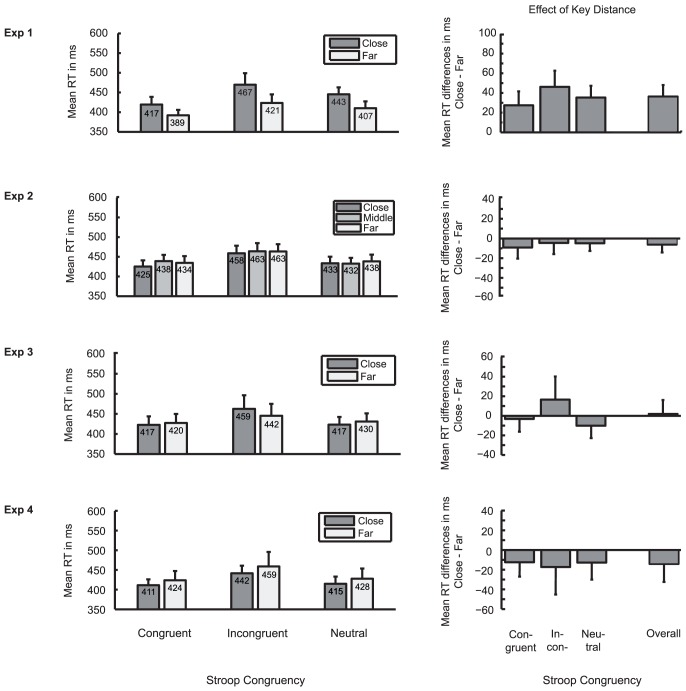
Results of Experiments 1–4 (all trials). Left column: mean reaction time (RT in ms) as a function of key distance (close, far) and Stroop congruency (congruent, incongruent, neutral) for all trials. Error bars represent SEM between participants. Right column: effect of key distance defined as mean RT difference between close and far conditions, for each Stroop congruency (congruent, incongruent, neutral), and averaged across all congruencies. Error bars represent SEM of mean close-far differences between participants. For the correct interpretation of these error bars see Franz and Loftus [24].

Fig. 2 and Table 1 display RT and error data, respectively. Error rate did not show any significant main or interaction effects of the above factors (all *p*≥.098).

**Table 1:** Error rate (M, SD in %) in Experiments 1–4 as a function of key distance (close, far) and Stroop congruency (congruent, incongruent, neutral).

		Congruent	Incongruent	Neutral
Experiment 1	Close	2.0 (3.4)	3.8 (4.3)	2.0 (3.0)
	Far	2.5 (3.8)	4.5 (5.6)	3.8 (6.7)
Experiment 2	Close	2.4 (3.4)	3.2 (2.7)	2.8 (2.9)
	Middle	3.2 (4.4)	3.8 (4.3)	3.4 (4.9)
	Far	3.5 (4.8)	3.3 (3.6)	3.0 (3.0)
Experiment 3	Close	2.3 (3.4)	6.8 (8.2)	3.3 (4.7)
	Far	2.8 (4.1)	7.3 (6.2)	4.0 (4.8)
Experiment 4	Close	3.4 (2.9)	6.1 (7.7)	2.9 (4.5)
	Far	2.9 (3.8)	5.3 (5.6)	4.7 (7.0)

For RT, a main effect of Stroop congruency was obtained (*F*(2,38)  = 8.84, ε = .62, *p* = .004, η_p_^2^  = .32). Responses were slower in incongruent than in congruent conditions (*t*(19)  = 3.73, *p* = .001, *d* = 0.83; *M* = 444 vs. 403 ms, 95% CI [17.85, 63.66]). The main effect of key distance was also significant (*F*(1,19)  = 9.67, *p* = .006, η_p_^2^  = .34): participants responded slower with close than with far keys (*M* = 442 vs. 406 ms, 95% CI [11.66, 60.55]). Stroop congruency and key distance did not interact significantly (*F*(2,38)  = 0.88, ε = .87, *p* = .413, η_p_^2^ = .04; Stroop effect for close vs. far keys  = 50 vs. 32 ms, 95% CI [−6.77, 44.2]).

#### 2.2 Analysis of first 30 trials per key distance

When we considered only the first 30 trials in each key distance block, error rate did not show any significant effects (all *p*≥.094). RT showed a main effect of Stroop congruency (*F*(2,38)  = 9.47, ε = .73, *p* = .002, η_p_^2^  = .33), with slower responses in incongruent than in congruent trials (*t*(19)  = 4.26, *p*<.001, *d* = 0.95; *M* = 450 vs. 404 ms, 95% CI [23.59, 69.18]). There was a main effect of key distance (*F*(1,19)  = 7.24, *p* = .014, η_p_^2^  = .28; *M* = 450 vs. 408 ms for close and far keys, 95% CI [11.30 71.83]), but no interaction between Stroop congruency and key distance (*F*(2,38)  = 0.17, ε = .71, *p* = .775, η_p_^2^ = .01; Stroop close vs. far  = 62 vs. 47 ms, 95% CI [−24.05, 52.26]).

### 3. Discussion

The RT pattern in Experiment 1 confirms the finding of a KDE in the Stroop task. In contrast to Lakens et al. [3] and Proctor and Chen [4] (Experiment 1), key distance affected RT similarly in both congruent and incongruent trials: participants responded generally faster when response keys were far apart than when keys were close together. Although the numerical size of the Stroop effect was somewhat smaller with far (incongruent-congruent  =  32 ms) than with close keys (50 ms), the interaction was not significant. Likewise, in the second of their three experiments, Proctor and Chen [4] (Experiment 2) found a significant main effect, but no interaction between key distance and Stroop congruency. However, there was a smaller Stroop effect in the far than in the close key condition. In the third experiment [4], key distance had no significant effect on RT, but again the Stroop effect was numerically smaller with far than with close keys. Our data is consistent with Proctor and Chen's [4] assumption that physical key distance enhances discriminability of responses, as reflected in faster responses with far keys. Results do, however, not suggest that higher response discriminability with far keys also attenuates the Stroop effect. In fact, a facilitation in both Stroop conditions, rather than only in incongruent trials, seems even more consistent with the explanation favored by Lakens et al. [3]: if humans have a cognitive tendency to represent mental categories in space, which is prompted automatically by perceived separation of response keys, then a KDE across all conditions of a classification task would be plausible. As to the time-course of the influence of key distance, converging results from analyses both on all and on initial trials of Experiment 1 do not suggest a decay over time like in Proctor and Chen [4].

Importantly, while results from Experiment 1, Lakens et al. [3] and Proctor and Chen [4] confirm that physical key separation has an influence on Stroop performance, they do not yet permit to infer whether this influence is automatic. First of all, displaying SR mapping instructions in a spatial configuration on the screen might bias participants towards encoding the color categories on the horizontal spatial dimension. Also, key separation was confounded with two other characteristics of the keys on the standard keyboard. Close keys were both labeled with letters (‘K’ and ‘L’), as opposed to far keys labeled with a letter versus a number (‘S’ and ‘5’; Fig. 1A). In addition to that, close keys were both located in the main typing area of the keyboard, while far keys were located in distinct parts, that is, ‘S’ in the main typing area, but ‘5’ in the numeric keypad which is visually separated from the main block via the spatial arrangement of keys. Hence the findings obtained with this response set cannot rule out that key separation is not the only critical factor in the response configuration for the KDE. Moreover, in the original set-up, key distance was varied in a narrow range at only two levels, therefore potential boundary conditions of the effect regarding absolute and relative distances between keys could not be tackled.

## Experiment 2

With Experiment 2 we designed a stricter test of the automaticity of the KDE: we refrained from displaying the SR mapping instructions during the experiment. We used un-labelled response keys with high measurement accuracy, adjusted to three different horizontal distances of a wide range. Furthermore, we controlled for effects of expectancy by drawing stimulus onset asynchronies from a memoryless distribution, rather than using a fixed interval as before. We counterbalanced SR mapping instructions within participants to control for order effects, and further enhanced the reliability of our measurement by presenting three times as many trials as in the original design. If key separation automatically drives the KDE, the effect should arise even under these conditions.

### 1. Methods

#### 1.1 Participants

Thirty-six participants took part in the experiment (22 females; age 18–53 years, *M* = 26.4).

#### 1.2 Apparatus, stimuli and procedure

Apart from the above mentioned modifications in response device, instruction, timing and number of trials and block order, we used the same basic task as in Experiment 1. Participants were instructed about the SR mapping before testing, and instructions were not presented along with the experimental stimuli. We used custom-made response keys without labels that were steplessly adjustable to various distances. RT was measured by means of a high precision DT9812-USB module (Data Translation, Inc., MA, USA). RTs registered by the Matlab-controlled DT9812 were on average only 3.6 ms larger than the latencies measured by a calibrated digital oscilloscope and showed low variability (*SD*  =  0.95 ms), thus very little noise was added to our measurement. In contrast, conventional keyboards were found to exhibit total delay times of 11 to 73 ms (*SD*  =  0.8–9 ms) [20].

Participants underwent 360 trials in six blocks of 60 trials each (20 per Stroop congruency: congruent, incongruent, neutral). We used three different key distances (close: 6 cm, middle: 51 cm, far: 108 cm measured from center to center; Fig. 1B) and two different SR mapping instructions (blue  =  right key, blue  =  left key). Key distance changed every two blocks and instructions changed every single block within each participant. Block order was counterbalanced between participants. Each block was preceded by 10 practice trials. A single trial started with a fixation cross in the center of the screen, presented for a fixed duration of 900 ms and an additional random duration drawn from a non-ageing exponential distribution (λ = 100 ms). Thereafter the target word appeared for 2000 ms, during which the response was made. Feedback (‘Falsch’, German for ‘wrong’) was displayed only after an incorrect response.

### 2. Results

#### 2.1 Analysis of all 360 trials

Fig. 2 and Table 1 display RT and error data. Error rate did not show any significant effects (all *p*≥.289).

For RT, a main effect of Stroop congruency was obtained (*F*(2,70)  = 30.5, ε = .77, *p*<.001, η_p_^2^ = .47), with longer responses in incongruent than in congruent trials (*t*(35)  = 6.27, *p*<.001, *d* = 1.04; *M* = 461 vs. 432 ms, 95% CI [19.62, 38.41]). The main effect of key distance was not significant (*F*(2,70)  = 0.67, ε = .97, *p* = .511, η_p_^2^  = .02; *M* for close, middle, far keys  = 439, 445, 445 ms, 95% close vs. far CI [−18.41, 5.96]). Again, Stroop congruency and distance between response keys showed no interaction (*F*(4,140)  = 0.74, ε = .83, *p* = .543, η_p_^2^  = .02; Stroop close vs. far  = 34 vs. 29 ms, 95% CI [−15.31, 24.55]).

#### 2.2 Analysis of first 30 trials per key distance

In the initial trials, error rate showed a main effect of Stroop congruency (*F*(2,70)  = 3.75, ε = .87, *p* = .035, η_p_^2^ = .1): participants made more errors in incongruent than in congruent trials (*t*(35)  = 2.33, *p*<.026, *d* = 0.39; *M* = 3.5 vs. 2.3%, 95% CI [.16, 2.34]). No other effects were significant (all *p*≥.247)

RT showed a main effect of Stroop congruency (*F*(2,70)  = 6.64, ε = .91, *p* = .003, η_p_^2^ = .16), with slower responses in incongruent than in congruent trials (*t*(35)  = 2.43, *p*<.020, *d* = 0.4; *M* = 451 vs. 428 ms, 95% CI [3.72, 41.52]). There was no main effect of key distance (*F*(2,70)  = 0.58, ε = .94, *p* = .553, η_p_^2^  = .02; *M* for close, middle, far keys  = 429, 436, 438 ms, 95% CI close vs. far [−28.33, 10.02]), and no interaction between Stroop congruency and key distance (*F*(4,140)  = 1.57, ε = .83, *p* = .195, η_p_^2^  = .04; Stroop close, middle, far  = 28, 39, 5 ms, 95% CI close vs. far [−13.13, 60.25]).

### 3. Discussion

After controlling several possible mediators of the KDE other than perceived key separation in Experiment 2, that is, without SR mapping instructions on the screen and with un-labeled response keys at three different distances, we found no indication that key distance affected performance. Results from the early trials corresponded to those from all 360 trials, ruling out that a KDE might have been present only at the beginning of the experiment. Effects of Stroop congruency in the first 30 trials were consistent in both error rate and latency, and therefore not suggestive of a speed-accuracy tradeoff in performance.

Albeit results of Experiment 2 confirm that horizontal key separation alone cannot cause the KDE, the comparison between Experiment 1 and 2 does not permit a definite conclusion which factors are actually responsible for the effect: on the one hand, an interaction between the configurations of stimuli and responses might lead to the KDE. A prerequisite for this interaction might be the addition of SR mapping instructions to the stimulus configuration, meaning that no effect was found in Experiment 2 because no instructions were displayed. On the other hand, horizontal key separation in Experiment 1 was confounded with labeling and spatial grouping of keys on the standard keyboard. Therefore, our results not yet ruled out that characteristics of the response configuration might be sufficient to elicit the KDE: labeling or grouping of keys, either by themselves or in a combination with horizontal key separation.

## Experiment 3

We performed Experiment 3 to exclude the possibility that it was the use of un-labelled and un-grouped response keys rather than the omission of the SR mapping instructions that actually cancelled the KDE in Experiment 2. We again adopted Lakens et al. [3] set-up and used an ordinary keyboard, as in Experiment 1. But we removed the SR mapping instructions from the screen, paralleling Experiment 2. If SR mapping instructions are crucial for the KDE, then we would expect no KDE in Experiment 3.

### 1. Methods

#### 1.1 Participants

Twenty participants took part in the experiment (19 females; age 19–26 years, *M* = 21.5).

#### 1.2 Apparatus, stimuli and procedure

The apparatus and stimuli were the same as in Experiment 1. The only difference in the procedure was that we instructed participants beforehand about SR mappings and did not display instructions throughout the experiment.

### 2. Results

#### 2.1 Analysis of all 360 trials

Fig. 2 and Table 1 display RT and error data. Error rate showed a main effect of Stroop congruency (*F*(2,38)  = 6.52, ε = .76, *p* = .008, η_p_^2^  = .26): participants made more errors in incongruent than in congruent conditions (*t*(19)  = 3.14, *p* = .005, *d* = 0.7; *M* = 7 vs. 2.5%, 95% CI [1.5, 7.5]). The main effect of key distance was not significant, as was the interaction between key distance and Stroop congruency (both *p*≥.413).

The RT analysis revealed a main effect of Stroop congruency (*F*(2,38)  = 5.01, ε = .69, *p* = .024, η_p_^2^  = .21), with slower responses in incongruent than in congruent trials (*t*(19)  = 2.83, *p* = .011, *d* = 0.63; *M* = 450 vs. 418 ms, 95% CI [8.25, 54.97]). RT showed neither a main effect of key distance (*F*(1,19)  = 0.00, *p* = .983, η_p_^2^  = .000; *M* for close vs. far keys  = 430 vs. 430 ms, 95% CI [−27.46, 26.7]) nor an interaction between Stroop congruency and key distance (*F*(2,38)  = 1.46, ε = .64, *p* = .245, η_p_^2^  = .07; Stroop close vs. far  =  42 vs. 22 ms, 95% CI [−15.4, 55.08]).

#### 2.2 Analysis of first 30 trials per key distance

In the early trials, error rate did not show any significant effects (all *p*≥.171). RT showed a marginally significant main effect of Stroop congruency (*F*(2,38)  = 3.47, ε = .77, *p* = .055, η_p_^2^  = .16): participants responded slower in incongruent than in congruent trials (*t*(19)  = 2.42, *p* = .026, *d* = 0.54; *M* = 448 vs. 417 ms, 95% CI [4.16, 57.1]). There was neither a main effect of key distance (*F*(1,19)  = 0.11, *p* = .739, η_p_^2^  = .006; *M* for close vs. far keys  = 427 vs. 425 ms, 95% CI [−32.2, 37.47]) nor an interaction between Stroop congruency and key distance (*F*(2,38)  = 0.67, ε = .67, *p* = .464, η_p_^2^  = .03; Stroop close vs. far  = 42 vs. 22 ms, 95% CI [−37.8, 77.43]).

### 3. Discussion

Consistent with Experiment 2, presenting only the Stroop stimuli on the screen in Experiment 3 cancelled the influence of key distance on RT that was found in Experiment 1. Analyses yielded the same results for the whole experiment and the initial trials. All trials considered both error rate and latency showed consistent effects of Stroop congruency, therefore not indicating a speed-accuracy tradeoff. Importantly, the combined interpretation of data from Experiments 2 and 3 now suggests that the SR mapping instructions are in fact a necessary part of the stimulus configuration on the screen concerning the KDE. Neither attribute of the response configuration, that is, horizontal separation, labeling, and grouping of keys, automatically elicits the effect. Of note, Nett and Frings [8] (Experiment 1a) recently tested the hypothesis that the KDE observed in Lakens et al.'s [3] original paradigm might be induced by the fact that both key labels in the close condition were belonging to the same category (i.e., letter), whereas in the far condition the keys were labelled with a letter and a number. They directly compared three participant groups who either used the same keys as in Lakens et al. [3], or keys that were consistently labelled with letters, or with numbers, in both distance conditions. They found an overall interaction between Stroop congruency and key distance, in addition to a main effect of key distance. Taking into account that the authors used an even smaller number of trials in their study (i.e., altogether 48, or only 24, according to the interpretation of the somewhat ambiguous design description) than the original experiment, which might have compromised the reliability of their measurement, this finding seems to rule out that the key labels are essential for the KDE. So far, our results indicate that perceived distance between response keys is only a necessary, but not a sufficient condition for the KDE. It seems that the effect emerges from an interaction between the response configuration and the stimulus configuration, the latter including both Stroop stimuli and SR mapping instructions.

Following Miller's [9] line of argument that the match between spatial stimulus information and spatial response codes strongly influences responses, it is conceivable that the KDE is generated by the actual correspondence between spatial positions of SR mapping information on the screen - as an equivalent to the spatial cues used by Miller - and spatial positions of the response keys. At first, the SR mapping instructions on the screen in Experiment 1 were in general spatially compatible with responses on the keyboard in front of the monitor. That is, the *relative* position of a correct response and the instruction about the correct key always coincided in terms of whether they were on the left or the right side of the set-up's midline. However, the degree of spatial correspondence between the *absolute* horizontal positions of SR mapping instructions and responses was actually higher with the far as compared with the close keys: using a computer monitor and a keyboard of both regular size, we presented SR mapping instructions at a distance of about 24 cm from each other (measured from center to center of the letter strings; see Fig. 1C). Far keys were separated by approximately 33 cm, close keys by 2 cm (center to center). Thus, higher spatial discriminability of wider separated responses might lead to response facilitation only if there is also better spatial correspondence between absolute horizontal locations of instructions and responses in the far key condition

Most studies of SR compatibility addressed spatial correspondence of stimuli and responses exclusively in relative terms of ‘left/right’ categories. We know of only few studies that manipulated absolute position in both stimuli and responses, the results of which suggest though that the correspondence of absolute positions may also influence performance: Stins and Michaels [11] (Experiments 1 & 2) studied fast reaching movements towards either a central or a more eccentric target (separated by approx. 15 or 65 cm), in response to one of six horizontally arranged visual stimuli. They found that RT was faster not only when relative positions of stimuli and response targets were compatible, but also when their absolute positions corresponded. Reaches directed at central targets were initiated faster in response to central stimuli than to more eccentric stimuli. Likewise, reaches towards eccentric targets were faster in response to eccentric stimuli than to central stimuli (both independent from the hand's starting position). When participants performed mouse key-presses instead of reaching movements in response to the spatial stimuli, the correspondence effect of absolute positions was evident again, however, restricted to eccentric responses. Furthermore, Adam et al. [12] varied the horizontal grouping of both the stimulus and response set in two of their response-cuing experiments (Experiments 2 & 3). They used all combinations of four possible stimulus and response positions that were arranged in either a left-right or inner-outer pattern. When both hands were used (the index and middle finger constituting the left-right pattern and the index and litter finger constituting the inner-outer pattern), results actually showed an effect of spatial stimulus-response compatibility on the set-level: RTs were shorter and accuracy was higher when stimulus and response configurations corresponded in their horizontal configuration. When only one hand was used (the left–right response set consisting of the thumb, index, ring, and little finger, the inner–outer response set consisting of the thumb, index, middle, and little finger), the compatibility effect was restricted to RTs with a short preparation interval of 60 ms. If eccentricity, or absolute position in general, were assumed a dimension which the stimulus and response set can share (in addition to relative left-right position), then the correspondence between spatial codes for stimulus and response eccentricity might influence performance similar to the correspondence between codes for relative positions; see [7].

## Experiment 4

Having established the connection between the stimulus configuration and the KDE in Experiments 2 and 3, Experiment 4 tested the idea that the influence of SR mapping instructions on behavioral performance might depend on the absolute spatial correspondence between instructions on the screen and the wider separated responses on the keyboard. Therefore, we presented both instructions at the center of the screen, so that the correspondence between the absolute horizontal locations of instructions and responses was no longer higher with far than with close keys. We kept relative spatial SR correspondence similar for both key conditions by presenting the instruction for the left key in the lower position and the instruction for the right key on top. In studies where the stimuli vary along a vertical and the responses along a horizontal dimension, response facilitation has often been shown for the mapping of ‘up’ to ‘right’ and ‘down’ to ‘left’ compared with the alternative mapping, suggesting orthogonal SR mapping preferences; see [21]. Thus, the relative position of a correct response and the instruction about the correct key would again be compatible in both key distance conditions, as in Experiment 1. However, the degree of correspondence between the absolute horizontal positions of SR mapping instructions and responses would now be higher with close keys than with far keys. Accordingly, we expected the KDE to disappear.

### 1 Methods

#### 1.1 Participants

Twenty participants took part in the experiment of which one was excluded because of an average error rate above 15% (*M* = 18.3%), leaving a sample of nineteen for analysis (16 females; age 19–27 years, *M* = 21.8 years).

#### 1.2 Apparatus, stimuli and procedure

Experiment 4 had the same design as Experiment 1, with the exception that now both SR mapping instructions were positioned centrally at the bottom of the screen, rather than to the sides (Fig. 1D). We presented the instruction for the left key in the lower position and the instruction for the right key on top.

### 2 Results

#### 2.1 Analysis of all 360 trials

Fig. 2 and Table 1 display RT and error data. Error rate did not show any significant effects (all *p*≥.084).

For RT, a main effect of Stroop congruency was obtained (*F*(2,36)  = 8.85, ε = .58, *p* = .005, η_p_^2^  = .33): participants responded slower in the incongruent than in the congruent condition (*t*(18)  = 2.77, *p* = .013, *d* = 0.64, *M* = 449 vs. 417 ms, 95% CI [7.55, 55.03]). The main effect of key distance was not significant (*F*(1,18)  = 0.57, *p* = .460, η_p_^2^  = .03; *M* for close vs. far keys  = 422 vs. 436 ms, 95% CI [−53.37, 25.24]). As before, key distance and Stroop congruency did not interact (*F*(2,36)  = 0.48, ε = .9, *p* = .940, η_p_^2^  = .003; Stroop close vs. far  =  30 vs. 35 ms, 95% CI [−45.86, 36.23]).

#### 2.2 Analysis of first 30 trials per key distance

In the initial trials, error rate did not show any significant effects (all *p*≥.069). For RT, a main effect of congruency was significant (*F*(2,36)  = 4.32, ε = .83, *p* = .029, η_p_^2^  = .19), with tendentially slower responses in incongruent than in congruent trials (*t*(18)  = 2.0, *p* = .061, *d* = 0.46; *M* = 467 vs. 436 ms, 95% CI [−1.54, 62.57]). There was no main effect of key distance (*F*(1,18)  = 2.29, *p* = .148, η_p_^2^  = .11; *M* for close vs. far keys  = 431 vs. 464 ms, 95% CI [−86.11, 20.28]) and no interaction between Stroop congruency and key distance (*F*(2,36)  = 1.90, ε = .74, *p* = .176, η_p_^2^  = .1; Stroop close vs. far  = 15 vs. 59 ms, 95% CI [−116.28, 27.81]).

### 3 Discussion

As hypothesized, when we presented the SR mapping instructions in the middle of the screen, wider key distance did not facilitate responses like in Experiment 1, where the instructions were displayed to the left and right. On the contrary, although not significant, our data showed slightly faster responses with close (423 ms) than with far keys (437 ms). The fact that modifying the position of the SR mapping instructions on the screen altered the influence of key distance strongly supports our assumption that the correspondence between the spatial configurations of responses and stimuli is an essential source of the KDEs as found by Lakens et al. [3], Proctor and Chen [4] and in our Experiment 1.

Positioning SR mapping instructions at the center of the screen even led to a slight facilitation with close as opposed to far keys, although there was no significant reversed KDE. Like in Experiment 1 the absolute positions of lateralized instructions corresponded better with far compared to close keys, in Experiment 4 now the absolute horizontal location of the instructions at the center of the screen corresponded better with the close keys at the center of the keyboard. However, the resulting advantage in absolute spatial SR compatibility that the close key condition had over the far key condition was probably not sufficient to facilitate responses with close keys significantly. That was because the responses with close keys were less discriminable than the responses with far keys. Moreover, due to the vertical arrangement of instructions there was an overlap between the absolute horizontal locations of both instructions and both close keys. The overlap might also have made response coding on the horizontal spatial dimension ambiguous in close key trials. This interpretation would be in line with findings of Proctor and Chen [4] who reported a KDE when displaying both SR mapping instructions in the right lower corner of the screen. According to our account, this configuration would reduce relative spatial SR correspondence to orthogonal compatibility between up-right and down-left positions as in the present Experiment 4. Also, none of the key sets would correspond perfectly with instructions in terms of their absolute locations. Thus, Proctor and Chen's [4] findings would be largely consistent with our notion that the horizontal configuration of instructions drives the influence of key separation. More precisely, higher spatial discriminability of farther separated keys accelerates responses only if the spatial SR configuration with far keys also provides higher SR compatibility than the configuration with close keys. In previous Stroop studies [3], [4] and our Experiment 1 both response discriminability and SR compatibility were higher in the far key condition, leading to a significant KDE. In our Experiment 4, compatibility was higher in the condition with close keys, however, response discriminability was lower with close than with far keys, and therefore no significant KDE emerged.

## General Discussion

Lakens et al. [3] reported that increasing the horizontal distance between two response keys in the color-naming Stroop task diminished the Stroop interference effect. They proposed that people automatically use perceived space between response keys to form internal spatial representations of stimulus categories, as an instance of a general tendency of extended cognition. Mental distance between stimulus categories then would facilitate classification of stimulus categories. Proctor and Chen's [4] investigations into these findings led them to conclude that wider key separation facilitates Stroop performance by way of increased spatial response discriminability.

However, we show that physical separation of response keys is by itself not sufficient to facilitate color-classification in the Stroop task.

### 1 Our results

In Experiment 1 we confirmed an effect of key distance on RT in Lakens et al. 's [3] Stroop task. In Experiments 2–4 we altered the stimulus configuration by manipulating the SR mapping instructions which had been presented on the screen. Effects of key distance were cancelled when SR mapping instructions were completely removed from the display in Experiments 2 and 3, and when both instructions were presented in the middle of the screen in Experiment 4.

Our two main findings are that necessary conditions for the KDE in the Stroop task comprise (1) displaying SR mapping instructions on the screen along with the Stroop stimuli, and furthermore (2) a spatial configuration of the SR mapping instructions which provides higher spatial compatibility with the better discriminable key set, in this case the wider spaced keys. The source of the KDE is thus not physical separation of response keys alone. Instead, the effect arises from an interaction between the configurations of both the stimuli and the responses.

Previous and present results altogether suggest that key separation facilitates responses in the Stroop task. There is, however, no consistent evidence for an attenuation of the Stroop interference effect. Separate analyses of our studies do also not confirm a transient KDE as reported by Proctor and Chen [4]. In the following we relate our findings to previous evidence from behavioral studies.

### 2 Response discriminability

Whereas we can demonstrate that the KDE is not due to response characteristics alone, results from earlier studies delineate the contribution of response discriminability to the influence of key separation. Extending Lakens et al. [3], Proctor and Chen [4] were able to exclude that either anatomical or spatial discriminability of effectors, rather than spatial discriminability of keys, added substantially to the KDE. Using the same basic Stroop task, they tested the influence of anatomical discriminability of effectors by contrasting three between-subject conditions. Participants pressed either (a) close or (b) far keys using the index fingers of the left and right hand, as in previous experiments, or (c) close keys using the index and middle fingers of the dominant hand [4] (Experiment 2). In a further experiment, the authors investigated spatial discriminability of effectors by letting participants operate the far keys with sticks held at a close spatial separation versus operating the close keys with sticks held far apart [4] (Experiment 3). The Stroop effect was of a similar size, regardless of whether participants responded with fingers from one or two hands, or whether they operated the keys with close or far apart sticks. The authors concluded that it was not discriminability of effectors, but rather spatial discriminability of response keys that facilitated response selection. Spatial distance between responses might also increases the salience of response coding on the horizontal spatial dimension, thereby enhancing response discriminability; see [22]. Evidence consistent with a predominant influence of spatial response separation on response selection came also from different domains, including spatial SR compatibility [10] and task-switching [23].

Proctor and Chen [4] attributed the KDE on Stroop performance to spatial discriminability of responses. Our results, however, imply that the spatial correspondence between positions of instructions on the screen and responses on the keyboard is another essential source of the KDE.

### 3 Interactions between stimulus and response coding

Our data show that the combination of the response and also the stimulus configuration is crucial to drive the KDE in the Stroop task. With the help of a theoretical framework by Adam et al. [12] we consider a new view into the Stroop paradigm that takes into account the role of the displayed SR mapping instructions in the emergence of the KDE.

In their theoretical account of results from response-cuing experiments, for example [9], Adam et al. [12] propose a ‘Grouping Model’ that emphasizes the interaction between coding of stimulus and response configurations: the evidence suggests an essential dominance of the spatial arrangement of visual stimuli in coding of responses. The organization of the responses, however, may provide constraints on the translation of visual information into the selective activation of a response, especially in cases where stimulus locations cannot be straightforwardly mapped onto the response configuration. Now, visual letter stimuli in the Stroop task are neither positioned spatially nor preceded by visual cues. Nonetheless, the above account offers a potential role of the displayed SR mapping instructions and their spatial locations: assuming that the instructions might, accidentally, have acted as cues, prompting the participant to code font colors in ‘left–right’ categories, this would result in spatial coding (or perceptual ‘grouping’) of these stimulus characteristics. According to Adam et al. 's [12] framework now, both the strength of grouping *within* stimuli and responses in terms of distinctiveness and also the match *between* the grouping of stimuli and responses determines the process of response selection. In the Stroop task, a larger physical distance between response positions would enhance the motoric grouping in the far key relative to the close key condition. Furthermore, our results make it plausible to assume that the more distinct motoric grouping with far keys might lead to a situation where the best correspondence between perceptual and motoric groups/codes arises in a set-up where the absolute positions of instructions on the screen coincide with the wider separated responses (Experiment 1) rather than with the close responses (Experiment 4). The resulting higher correspondence in the far key condition would lead to fast, automatic response selection, whereas response selection in the close key condition would draw upon slower, non-automated top-down-processes (in this case, the Grouping model would assume a process of reorganization of response codes that attempts to match the configuration of the stimulus codes). Importantly, our results strongly suggest that spatial coding of Stroop stimuli would not be established by default without the spatial organization of mapping information on the screen, even if the distance between response keys is varied. This view is supported by our main findings that no KDE on Stroop interference emerges when SR mapping instructions are not displayed and that the effect furthermore depends on the spatial position of instructions on the screen.

Very recently, Nett and Frings [8] (Experiment 1b) also took notice of the spatial relation between on-screen instructions and responses as a potential confound in the original paradigm. Their direct comparison between two subject groups who were, or else, where not presented with the lateralized SR mapping instructions in a Stroop paradigm similar to Lakens et al. 's [3] did not yield any group differences. A KDE was found across both groups, however, exclusively in error rates. Mean RT showed the expected numerical differences between Stroop effects in the group presented with SR instructions (close  =  19 ms, far  =  40 ms), while Stroop effects were similar without SR instructions (close  =  26 ms, far  =  28 ms). This finding seems to be not in line with those of our Experiment 3. It is neither self-evidently consistent with previous Stroop experiments, including Nett & Frings' [8] first conceptual replication, where main analyses revealed effects of key distance on RTs only. Whereas a potential shift of participants' speed-accuracy criteria would be compatible with the data from Nett and Frings' [8] two experiments, it would contradict Proctor and Chen's [4] suggestion that an alignment of speed-accuracy criteria between participants takes place over the course of an experiment. Nett & Frings [8] however, state that they presented only 24 and 30 experimental trials in their first and second experiment, respectively. Their design description is somewhat ambiguous concerning whether this denotes the trial number altogether per participant or per key distance condition (although they state to have used as many trials as Lakens et al. [3] in Experiment 1b, which would be 30 trials per distance condition, or 60 trials). In any case, even 48 and 60 trial repetitions would make an adjustment of speed-accuracy criteria unlikely. If they chose in fact less repetitions than Lakens et al. [3] and Proctor and Chen [4] (Experiment 1, and for analyses), this might at the same time challenge the reliability of the measurement itself. Until independent evidence corroborates Nett & Frings' [8] findings, we still see the present results of both our Experiments 3 and 4 as indicating an essential role of the spatial relation between mapping instructions on the screen and responses for the KDE.

### 4 The influence of key distance on performance in the Stroop task

Proctor and Chen [4] noted that an attenuation of the Stroop effect in the incongruent condition by increased physical distance between response keys would indicate that Stroop interference can be affected by discriminability of responses at the response-selection stage [15], thus the origin of interference might not be confined to the stage of stimulus encoding.

Summing up results from previous and present Stroop studies in which key distance was expected and found to affect performance, there is no conclusive evidence that this influence modifies the Stroop interference effect: so far, three studies [3], [4], [8] (Experiment 1 and Experiment 1a, respectively) found a significant interaction between key distance and Stroop congruency in RT which was due to less Stroop interference with far than with close keys. In three previous experiments [4], [8] (Experiments 2 & 3 and Experiment 1b, respectively), and our Experiment 1, the key distance-by-Stroop congruency interaction was not significant in RT, apart from similar RT patterns [4], or else an effect was present in error rate only [8] (Experiment 1b). Two experiments showed either a main effect of key distance alone [4] (Experiment 2), or in addition to an interaction [8] (Experiment 1a). Therefore, the evidence does not permit to conclude that key separation might suppress interference from the task-irrelevant stimulus characteristic (word meaning) on the classification of font color. The main effect of key distance in Experiment 1, with faster responses in the far key condition, would rather indicate that categorization performance is generally improved by a larger key distance, probably via faster response selection. Trivial explanations for a main effect in terms of motoric or postural mechanisms seem at least unlikely: in a control experiment by Lakens et al. [3], key distance did not affect performance when demands on response selection were minimized by having participants indicate the mere occurrence of either red or blue letter strings with alternately the left or right key. Of note, a main effect would even be more consistent with Lakens et al. 's [3] notion of automatic spatial representations than an interaction.

Furthermore, our results do not further support Proctor and Chen's [4] suggestion that the KDE on Stroop performance is transient and therefore manifest only in the early trials of an experiment. Because Lakens et al. [3] obtained their original results with 10 trial repetitions per Stroop congruency (incongruent, congruent, neutral) and key distance condition, which is an unusually small number of repeated measurements for RT experiments, Proctor and Chen [4] investigated whether the KDE is influenced by practice at the Stroop task. They compared analyses including the initial 60 trials (i.e., two blocks of 30 trials per key distance condition each) of their three Stroop experiments with analyses including all 720 trials from two of their experiments (as their first study was an exact replication of Lakens et al. it comprised only 60 trials). It turned out that the pattern of performance that indicated diminished Stroop interference with far apart keys could be obtained in the replication study and in the first 30 trials per key distance condition of the other two experiments (even though the corresponding interaction was significant only in the replication experiment), but not when all 720 trials were analyzed. The authors suggested that practice over the course of the experiment might have led to an improvement in the ability to discriminate the close keys. However, as we stated above, this notion would contradict Lakens et al. 's [3] idea of an automatic influence of key separation on mental representation. As an alternative explanation, Proctor and Chen [4] proposed that participants might have adjusted initially different speed-accuracy criteria for the close and the far key condition. This account was not clearly supported by their data, though, because there was no significant interaction effect on error rate. In the end, a transitory KDE that vanishes after only about 30 experimental trials would at the same time have much less implications for Stroop experiments and other categorization tasks in general, where the usual number of trials is much higher.

When we performed supplemental analyses including only the first 30 trials from each key distance condition in Experiments 1-4, we found ANOVA results to be largely consistent with our main analyses. RT showed a main effect of Stroop congruency in all experiments. Most importantly, there was a main effect of key distance in the first trials of Experiment 1, consistent with the analysis including all trials. Again in accord with our main analyses, we did not find an interaction between key distance and Stroop congruency in Experiment 1 or any of the other experiments. Thus, our supplementary analyses do not support the assumption that novelty of the task constitutes a boundary condition of the KDE on Stroop performance. In the end, however, any proposition concerning the nature of the influence of key separation on color categorization must remain tentative until future studies investigate possible effects of key separation on other cognitive tasks with a similar stimulus-response set-up.

## Conclusions

In the color-naming Stroop task, when participants have to indicate the stimulus color by pressing either a left or right response key, RT can be moderated by the physical distance between these keys, as was first reported by Lakens et al. [3].

Our main finding is that the influence of key distance on performance in the Stroop task depends on spatial characteristics of the unusual stimulus configuration in the paradigm used by Lakens et al. [3] that have not been considered so far: the distance between keys exerts an influence on RT only in conditions where (a) instructions about mapping of keys to colors are visible on the screen in addition to the actual Stroop stimuli, and (b) these instructions are displayed in positions that correspond with the positions of the wider separated keys on the horizontal dimension.

The present evidence contradicts Lakens et al. 's [3] proposal that perceived space between response keys automatically gives rise to spatial representations of color categories. In line with Proctor & Chen [4], we suggest that spatial response discriminability contributes to the KDE. Over and above their account, our findings indicate that the effect also depends on spatial SR compatibility, where visually displayed SR mapping instructions are working as a part of the stimulus configuration together with the Stroop stimuli. Higher spatial discriminability of wider separated keys seems to facilitate selection of the correct response, but only if there is spatial correspondence between SR mapping instructions and wider separated keys.

The present evidence altogether does not favor a specific reduction of color-word interference by increased key separation but rather an unspecific facilitation of responses irrespective of color-word congruency. Our findings do furthermore not support the assumption that the KDE is subject to practice.

Finally, current data does not seem to warrant Lakens et al. 's [3] conclusion that “even when there are no existing spatial metaphors for the concepts that are being categorized (i.e., there is no conceptual metaphor mapping blue and red onto the horizontal dimension), any two categories are automatically differentiated in space” (p. 890). Evidence from the employed Stroop task is not suitable to support their intriguing idea of a “basic human tendency to represent categorical differences in terms of spatial differences” (p. 891), because spatial characteristics of the stimulus configuration might bias participants to do so. Not until future evidence shows a KDE on classification of stimuli which clearly lack any spatial context, there are no general implications for the numerous findings obtained in similar experiments with spatially arranged keys.
